# Blockchain-based Reputation for Intelligent Transportation Systems

**DOI:** 10.3390/s20030791

**Published:** 2020-01-31

**Authors:** Liviu-Adrian Hîrţan, Ciprian Dobre, Horacio González-Vélez

**Affiliations:** 1 Faculty of Automatic Control and Computers, University Politehnica of Bucharest, 060042 Bucharest, Romania; ciprian.dobre@cs.pub.ro; 2 Cloud Competency Centre, National College of Ireland, D01 Y300 Dublin, Ireland; horacio@ncirl.ie

**Keywords:** Intelligent Transportation Systems, blockchain, trust, reputation, privacy, smart cities, mobile crowdsensing

## Abstract

A disruptive technology often used in finance, Internet of Things (IoT) and healthcare, blockchain can reach consensus within a decentralised network—potentially composed of large amounts of unreliable nodes—and to permanently and irreversibly store data in a tamper-proof manner. In this paper, we present a reputation system for Intelligent Transportation Systems (ITS). It considers the users interested in traffic information as the main actors of the architecture. They securely share their data which are collectively validated by other users. Users can choose to employ either such crowd-sourced validated data or data generated by the system to travel between two locations. The data saved is reliable, based on the providers’ reputation and cannot be modified. We present results with a simulation for three cities: San Francisco, Rome and Beijing. We have demonstrated the impact of malicious attacks as the average speed decreased if erroneous information was stored in the blockchain as an implemented routing algorithm guides the honest cars on other free routes, and thus crowds other intersections.

## 1. Introduction

With the mass adoption of mobile devices, both industry and academia have focused on developing a new concept, namely, *mobile crowdsensing*. Mobile crowdsensing enables continual collective perception by seamlessly employing the sensors, processors and storage from mobile devices such as smartphones, wearable devices, tablets, and others [1]. These communication and sensing patterns differ from those of classic processing and storage equipment, as they produce near-continuous data streams with intermittent peaks and troughs at different data rates. Consequently, there are numerous research works to adaptively improve the collection, storage, and uploading of data in the mobile crowdsensing domain.

By securely interconnecting diverse heterogeneous data sources and multiple technologies, smart cities integrate homes, street furniture, utility grids, energy storage, and transportation and traffic information management systems into a functioning ecosystem to make their infrastructure and services more accessible to citizens and, ultimately, improve quality of life. By extending transportation and traffic information management systems with innovative computer-enabled information services, *Intelligent Transportation Systems* (ITS) enable users (“travellers”) to smartly choose their transportation and, ultimately, make cities safer, more coordinated, and energy efficient. ITS are a major and indispensable component of any smart city these days.

ITS focus on reducing travel times, fuel consumption and pollution, as well as improving traffic safety. Typical innovative ITS applications rely on the Internet of Things (IoT)—the global network of interconnected physical artefacts—and include car navigation and traffic voice directions systems, municipal/regional traffic signal control systems, electronic message/notice boards and automatic number plate recognition among other. In terms of number of users, by far the most widely-used applications are car navigation systems.

Nonetheless, ITS come with a series of constraints mostly related to privacy preservation of users. Privacy preservation represents a major concern, taking into account the large volumes of data and the current data protection regulations, e.g., General Data Protection Regulation in Europe. The user identity must be protected so, first of all, all systems should provide a series of available policies whereby users can choose which data they share with the system. Also, the analysis of the data created by the devices and manipulated by other untrusted entities should not reveal personal information or any pattern through which it can be identified.

By using blockchain technologies to address IoT privacy and security [2], we propose to integrate travellers and public transport as a transportation continuum, to develop a secure reputation system where users transmit traffic information and receive travel information which can relied on. The reputation is based on traveller behaviour and their history regarding the type of data provided to the system. The system determines the traffic conditions within the areas from which the data is transmitted and can redirect users to the fastest routes according to the preferred means of transport. Both reputation and valid traffic data are permanently stored in a tamper-proof blockchain which cannot be modified by attackers. Data validity is established by travellers within the same cluster/geographical area via a consensus mechanism that takes into account the reputation of participants.

Widely considered immutable time-stamped data structures, *blockchains* implement peer-to-peer networks where participants can verify interactions concurrently using decentralised peer-to-peer consensus protocols. As an emerging technology trend, different research [3,4] and industrial [5] perspectives have been assembled to document its potential disruptive impact [6].

Blockchains have five unique characteristics:

1. Peer-to-peer communication without a central authority.
2. Transparent transaction processing with optionally-disclosed ownership.
3. Decentralised transaction history verifiable by all participants.
4. Immutability of records assuring chronological sequence and accessibility.
5. Logic-based processing to trigger algorithms and events.

The aforementioned characteristics have made blockchain particularly suitable to manage cryptocurrencies: electronic cash systems administered via peer-to-peer consensus. Indeed, the most widely known for cryptocurrency, the Bitcoin [7], remains somehow the gold standard for financial blockchain applications. Nonetheless, although blockchains have been used extensively in financial entities, their decentralised immutability characteristics have made them particularly suitable for applications in other domains as diverse as Law [8], Food Traceability [9], Open-source Software Management [10] and indeed IoT [11].

Blockchains can be construed as distributed databases in which all member-approved transactions are permanently stored, as a result of a consensus algorithm. The transactions are stored within blocks, in chronological order. The number of blocks is potentially unlimited, and the impossibility of modifying them is realised through the implemented algorithms. The hash stored within each block is proof of the fact that the blocks are ordered and unmodified. The consensus algorithm is the process through which the distributed network reaches an agreement on the transactions and blocks validity. The design of consensus algorithms also takes into account the fact that in the network there are unreliable nodes, processes or services may become unavailable, or communication may be interrupted. An agreement can be reached with more than 50% of nodes within the network.

This paper is organised in the following sections. Section 2 introduces an overview of related works. Section 3 describes the proposed system design, including a detailed account of the applied transactions. Section 4 assembles a privacy and security analysis, followed by Section 5, where we present our experimental results. Finally, Section 6 enumerates some conclusions and future directions for our work.

## 2. Related Work

This section presents the current state-of-the-art regarding establishing a reputation in ITS as well as proposals for the implementation of reputation systems using blockchain. We have also considered proposals on the improvement of the medium access control protocol in the wireless access vehicular environment as well as new methods to ensure the security and confidentiality in the blockchain. Finally, works on blockchain technology in reputation systems for ITS are analysed.

In [12], the authors model pairwise trust and system-wide reputation of crowd contributors using a linear algebra approach. With the aim of fostering explainability and auditability, their system stores the contributor models as smart contracts in a private Ethereum network, and then implements a recommendation and explanation engine based on the stored contributor trust and reputation smart contracts.

In [13], a reputation system is proposed based on blockchain technology with the purpose of solving the current generation reputation systems. Each user creates a positive transaction if he/she has received the requested file signed with the sender’s private key, or a negative transaction. The positive transaction consists of the reputation score, the timestamp and the hash of the received file. The miners verify the validity of the transaction by contacting each entity involved in the process and requesting a signed proof consisting of file hash and a random nonce sent by the miner to be included. The miners assemble the verified transactions in a block. Among the methods to prevent the creation of multiple identities, the authors propose to link the users with the IP address or with the email address. Reputation is not stored in the blockchain; the clients calculate the reputation of other nodes by themselves.

In [14], the authors propose a solution to certify the reputation of a member of the web community on top of the Bitcoin blockchain. After purchasing a particular service or product, the vendor creates a voucher transaction with a voting fee and an incentive. This transaction is sent to the customer to be co-signed. The consumer has the possibility to sign the voucher, which implies the validation of the contract execution. Thus, the producer’s reputation grows. A disadvantage of this system is the Sybil attack. An attacker can create fake consumer identities that will sign voucher transactions. As the voting fee is a percentage of the price, free services or products are affected.

A publisher–subscriber reputation system based on Bitcoin is presented in [15]. The model is comprised of publishers, sensors, and subscribers. Publishers collect data using sensors and publish a topic. The subscribers suggest their intention to subscribe using the ElGamal encryption scheme and make a deposit. The publishers establish a correspondence between the requests and the topic, encrypt the data with a symmetrical key, and send the encrypted text to the subscriber. In addition, their role is to send this match to the Bitcoin system. The subscriber receives the notification and pays the publisher. In the end, the publisher reputation is up to date and can receive bitcoins.

### 2.1. Trust and Reputation in Vehicular Systems

Trust and reputation management in vehicular ad hoc networks (VANETs) is a crucial matter, as broadcasted messages that represent traffic events should be endorsed by drivers within the same area and rebroadcasted or are discarded if the trust value is less than a predefined threshold. The overarching goal of trust in VANETs is to detect dishonest peers and their malicious data and, eventually, award incentives to honest peers discourageing self-interested behaviour [16]. A user reputation takes into account all endorsees made by other users, as well as a historical factor. Even though a vehicle may have a pseudonym as an identifier, systems should not provide a mechanism for changing it and, thus, following certain patterns, the identity should not be revealed.

In [17], a system for certified reputation is described, where vehicles communicate using digital certificates that contain the reputation level. The design includes additional entities, such as static communications infrastructure units called Road Side Units, Governmental Transportation Authority, Certification Authority and Central Control of Traffic Operation. Central Control of Traffic Operation is responsible to check the events’ validity, taking into account the information provided by other vehicles, and to compute the vehicles’ reputation. The vehicles are able to communicate directly and check the reputation that is stored and transported using a hardware security module.

In [18], the authors present the main wireless access vehicular environments, namely, IEEE 801.11p and IEEE 1609.4, as well as some proposals regarding the improvement of IEEE 1609.4 multichannel operation. The aforementioned research studies improve some performance parameters, but also have some limitations. The VANET environment has as main components onboard units (OBUs), electronic equipment installed in vehicles, and roadside units (RSUs), electronic equipment installed on the roadsides having the role of communicating with OBUs. With the aid of these devices, vehicle-to-vehicle (V2V) and vehicle-to-infrastructure (V2I) communication are achieved. The authors analyse each of the works, also presenting improvement solutions without causing the performance to decrease.

An innovative method of protecting data privacy and avoiding collision attacks using blockchain is presented in [19]. The blockchain network acts as a proxy server or as a trusted authority. Its role is to register participants and generate pairs of public and private keys, to verify access to the data stored in the cloud in accordance with the encryption key and related security policy, as well as to re-encrypt information if secondary users want to access the data. Also, the blockchain network has the role of generating a contract to verify the use of data, data that is stored encrypted in a cloud server.

In [20], the authors have presented a communication mechanism that provides trustworthiness for vehicles behaviour based on a reward system. The blockchain is used to store details, such as reputation or public keys, about every vehicle within the system. Those have an encrypted unique number issued by trusted entities such as vehicle sellers or authorised dealers. The reputation is earned by calculation of some computation within the group communication, although it is not used in route computation. At the same time, the reputation of the vehicles does not diminish if they provide false information. Given that vehicles communicate directly with other vehicles in the same geographical area, and their reputation is calculated by other traffic participants, we can conclude that an attacker can make a match between the real identity of a driver and his/her blockchain identity. The consensus protocol is based on Proof of Driving, where the broadcasted message is validated if the verification is passed by more than 50% of the cars within the same network. These authors have also presented in [21] a trust environment based on intelligent vehicle framework. The proposed mechanism uses three main technologies: communication network, Vehicular Cloud Computing and blockchain technology. Blockchain architecture has seven layers; it also includes trust and privacy mechanisms.

In [22], the authors present a reputation system based on blockchain technology where the cars rate the messages based on their own observations. The system is comprised of four types of entities: trusted authority with the role of vehicle registration and capacity certification, ordinary vehicle, malicious vehicle and miner. The vehicles have the ability to broadcast traffic data, verify the credibility of messages received based on the issuer’s reputation and generate a rating. Miners are selected from vehicles and their role is to create a block with all the ratings and to send it to all the vehicles. Those check the block and if it is accepted, it is added to the blockchain. The blockchain contains only validated ratings by other traffic participants. The presented system does not save traffic information, and privacy-preserving methods are not presented.

In [23], a blockchain-based anonymous reputation system is presented. The following main actors are part of the system; the Certificate Authority (CA), the Law Enforcement Authority (LEA) and the vehicles. The authors use a blockchain for certificates, a blockchain for revoked public keys and a blockchain for messages. The communication between vehicles is performed directly, in an encrypted manner using a public–private key system. Although only the LEA knows the correspondence between a public key and the real identity of a user, an attacker might figure out it by analysing the broadcasted messages within the network and his/her physical presence in certain locations.

The authors of [24] describe a reputation-based blockchain mechanism to secure the cache in the vehicular environment and enhance the trust between cache stored and consumer vehicles. Each block contains, among others, the following fields; the name of the provider who served the data, the name of the data validator that updated the provider’s reputation and the name of the content. Through the peer-to-peer network, when a node requests information, data is forwarded between all intermediate nodes. Each of the intermediate nodes queries the blockchain asking about the provider’s reputation. Through this method, the authors ensure that the data is secure, but the disadvantage is the generation of a large number of messages within the network. Finally, after consuming/validating the received data, the consumer node creates a new block informing the entire peer-to-peer network about its validation.

A traffic event validation and trust verification mechanism are presented in [25]. The authors propose the use of the Proof of Event mechanism in two-pass validation. Initially, the data is collected by Roadside Units (RSUs) and broadcasted to vehicles within the same area. If those validate the data within a predefined period, RSU adds to the local-chain the traffic event, along with the signatures of the vehicles that validated them. The system includes a local-chain maintained by RSUs within the same area and a global-chain updated over a longer period. Such a system is difficult to be implemented because the installation and interconnection of RSUs over a large geographical area are quite expensive. Also, the system does not take into account the reputation of vehicles and their history. Thus, in a geographic area where a fake traffic event is guaranteed by three malicious vehicles and invalidated by a vehicle with a good history, the event will be validated.

### 2.2. Contribution

During our research, we realised that establishing reputation and maintaining confidentiality at the same time in blockchain-based ITS solutions is at an early stage. The various literature reviewed in these sections has some shortcomings. For example, there are proposals that establish trust in the system without taking into account privacy, other proposals do not use revealed trust in establishing future transactions and other proposals are difficult to be implemented in real-life. Our paper addresses these shortcomings that can be found in the specialised literature, providing a system design that guarantees trust in ITS while ensuring the confidentiality of the users’ identity. Table 1 shows a comparative analysis following the advantages and disadvantages of the proposed solutions using the blockchain technology in reputation systems implemented on ITS.

## 3. System Design

In this paper, we present the design of a reputation system based on blockchain technology and applied in Intelligent Transportation Systems. Users have the possibility to receive real traffic information and, in turn, contribute with data to the system to have a common sense of traffic. Based on their behaviour and, more precisely, the veracity of the information provided, the system takes into account a very important variable, namely the users’ reputation. Depending on the users’ reputations that contributed with data, the design is able to make a final decision regarding the truth about it. Traffic data cannot be affected by attackers using bots or fake emulated entities, and reputation or identities cannot be modified or artificially inserted into the immutable ledger. Depending on the information received, the software solution provides to customers useful and reliable traffic information, such as the route from the start location to end location within a city. In the following, we will present the design of such a system, following the main components of blockchain technology.

**Figure 1:**
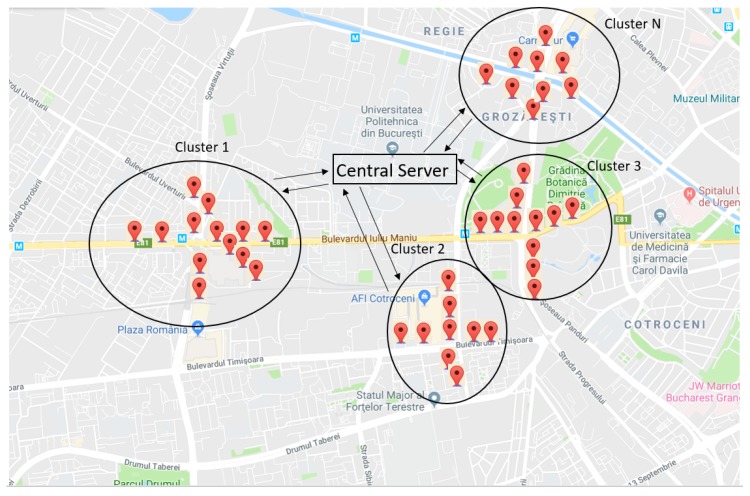
Geographical areas representing clusters.

The system includes two main actors: the users and the central server. The entire system contains information such as traffic data, the reputation of enrolled users, and related maps. Customers are grouped in clusters represented by geographic area, as shown in Figure 1, and, after the validation within the consensus algorithm, blockchain is used to store traffic events as well as the reputation of the users. The system uses the benefits of this technology such as immutability and security of stored data in order to provide unaltered and reliable information to users. In the following, we present the technical details, taking into account the main components of blockchain technology.

**Figure 2:**
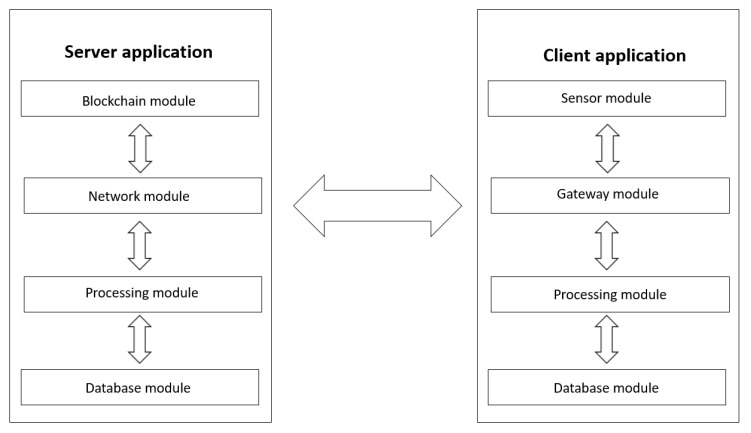
System design.

The proposed software solution involves the existence of two main actors: the users and the central server, represented by the corresponding software applications as shown in Figure 2.

Users are represented within the system through the client application and are grouped according to their location, more exactly by latitude and longitude. The users have a set of security policies at the client application level, and thus have the option of sharing data or not, to exclude location sharing in some areas, to disable data sharing if the traffic speed is greater than a certain threshold, etc. They have the role to interrogate the server through the client application to get the optimal route from the starting location to the final location, using the best transport means. They also provide information regarding traffic, for example, traffic events or the availability of transport means. Also, they validate other events sent by users from the same cluster, during the consensus algorithm.

The main storage and processing unit is represented within the system by the server application. It is designed to provide customers the optimal route from the start location to the end location. It receives traffic data sent by users through the client application, identifies other clients in the same cluster/geographic area and interrogates them to validate the received traffic information. The server also has the role to calculate customer reputation, to save it into internal blockchain alongside information regarding traffic.

As shown in Figure 2, each of the two systems, server application and client application are divided into modules, each having a different and very important role in the whole.

**Figure 3:**
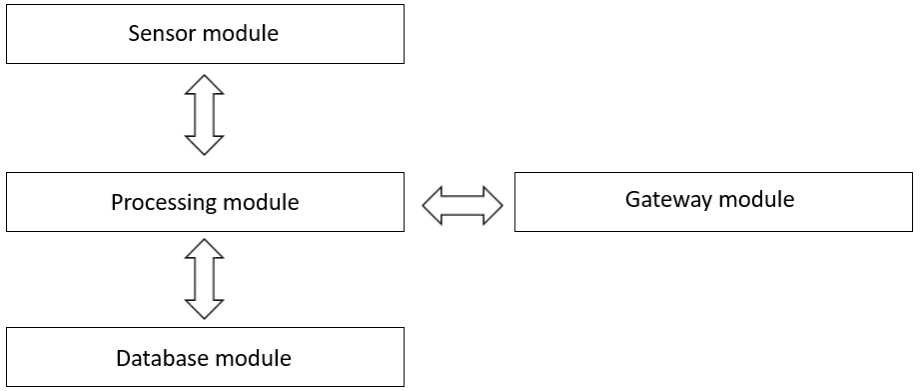
Server application system design.

The server application has the following modules, as shown in Figure 3.

**Figure 4:**
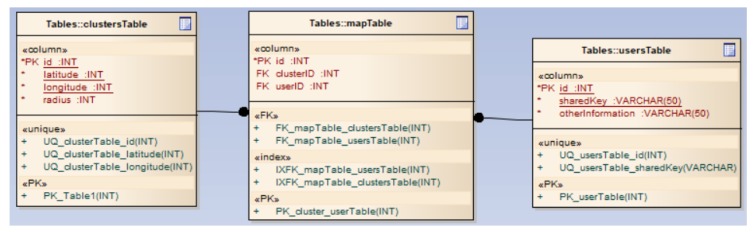
Database module-server application.

- Blockchain module: Stores blocks with user transactions, their reputation and traffic events. Traffic data is validated by users in the same cluster, whereas data regarding users and reputations are created by the server.
- Network module: Manages the interaction with users through client applications to receive/transmit route information, as well as to receive information about the availability of the means of transport. Also, this module has an important role in the consensus algorithm, as all members of the same cluster are interrogated regarding traffic events.
- Processing module: Enables the responses in real-time to user requests. It interacts with other modules within the server application and, through the network module, the processed information is transmitted to the clients. This module performs all processes, such as calculates the optimal route from start location to destination, the estimated duration of the travel, etc.
- Database module: Manages and stores the correspondence between users and clusters, which is employed within the consensus algorithm for querying clients in the same cluster or geographic area. Figure 4 shows an example of how this module is implemented.

**Figure 5:**
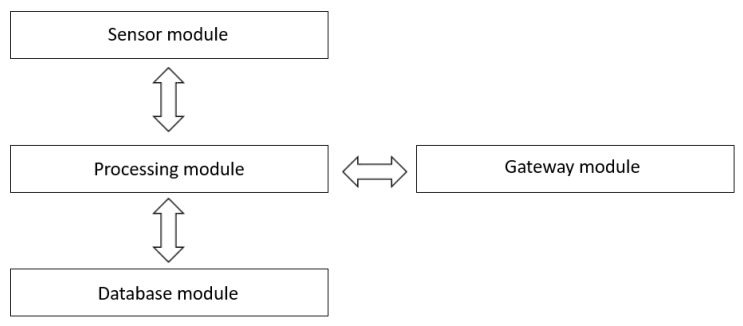
Client application system design.

As shown in Figure 5, the client application contains the following modules.

**Figure 6:**
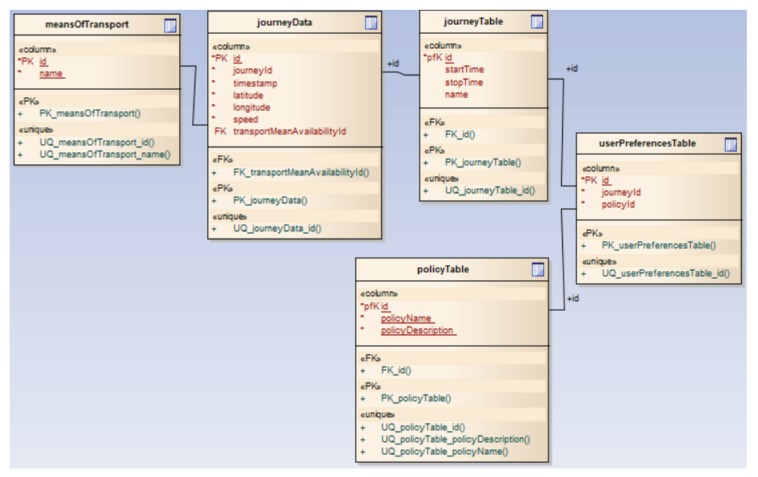
Database module – client application.

- Sensor module: Acquires all the necessary information (speed, location, user options, etc.) from various sensors (accelerometer, gyroscope, GPS, etc.) of the device on which the client application is installed.
- Processing module: Has the role of processing all the information received from other modules within the client application, as well as the information received from the server application.
- Database module: Stores data sharing security policies. Among those, we can list: apply/disable location sharing, apply/disable speed sharing, disable speed sharing if it exceeds a certain threshold, etc. Figure 6 presents an example of the database.
- Gateway module: Manages the interaction with the server application, i.e. to receive data such as the route and to transmit filtered data using the database module.

Our system design is based on blockchain technology, which through Bitcoin has demonstrated that it can solve a problem of trust in an unsafe environment. In Bitcoin, the nodes, represented by powerful processing units, invest a certain level of effort in an intense computational process to identify a solution to a proof-of-work puzzle. Data is stored in the blockchain and, once stored, it can never be modified until the entire computational process is restored. Each node within the network has a full copy of the blockchain that is downloaded when it joins the network and automatically syncs with the new blocks.

Our goal is to provide an ITS solution that is able to create secure transport routes based on a reputation system where the information comes from an unsafe environment. The nodes are represented by the client applications installed on users’ devices. The central server maintains a list of the identities of all the nodes. Those are identifiable within the network using unique ID associated with a unique encryption key. The nodes are divided into clusters or geographic areas and their role is to transmit information and validate other information from the same cluster. Each node has a reputation according to its history and they only communicate with the central server.

Communication between nodes in the same cluster is not direct but is done through the central server in order to validate a specific traffic event. Using symmetric encryption technology, the transmitted information is encrypted to ensure the confidentiality, integrity, non-repudiation and authentication.

**Figure 7:**
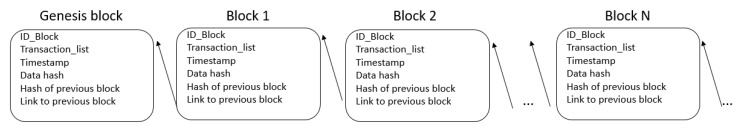
Blockchain.

Blockchain is stored within the central server and consists of blocks generated as a consequence of validated traffic events. Blockchain also takes into account the process of “ageing” the stored data. Thus, over time, if the number of nodes that invalidate the event grows, a new block will be stored in the blockchain where this new information is stored. Using the information found in the blockchain, the system is able to achieve the main objectives, such as computing the optimal routes and computing the users’ reputation. Taking into account that the information found in the blockchain is accessible to all users in terms of that any user can query the system regarding the optimal route between two locations, we can define the blockchain as open “permissionless”. A block contains data about a single event and, according to Figure 7, it contains the following fields: the ID of the block, the transactions list, the timestamp, the data hash, the hash of the previous block and the link to the previous block.

The consensus algorithm represents the main pillar of blockchain technology, through which it reaches reliability in a decentralised untruthful network. Through this process, a multitude of nodes within a peer-to-peer network reaches an agreement regarding a particular fact that leads to the creation of a new block within the blockchain. At the same time, the role of a consensus algorithm is to prevent the monopoly of nodes with intentions to create blocks containing false information.

The first consensus algorithm described is the Proof-of-Work (PoW) algorithm. In Bitcoin, proof-of-work has the role of processing transactions that should be introduced into the blockchain. The process of generating proof regarding the insertion of a new block within the blockchain is called mining, and the entities involved in the mining process are called miners. PoW involves solving complex mathematical computations, cryptographic puzzles before the information is added to the blockchain. The winning miner is rewarded in Bitcoin for the computational effort made as well as for auxiliary expenses. The difficulty of the process is dynamic: a new block is generated every 10 minutes. After the winning miner generates the new block, all other nodes start working on the next one.

Taking into account the entities that take part in the system design, the storage modality and the blockchain role, the geographical position of the participating nodes, we consider that the use of consensus algorithms such as Proof-of-Work is not justified because the participating nodes have limited hardware resources. In our system design, the validity of the information sent by the participants is guaranteed by the rest of the nodes located in the same geographical area in correlation with their reputation. The validity or invalidity of an event determines the creation of related transactions that contain data regarding the nodes’ updated reputation as well as data regarding the event. The list of transactions created during the consensus algorithm that refers to the confirmation or cancellation of a single event leads to the creation of a block that will be stored within the blockchain. Algorithm 1 describes this process in more detail.

Algorithm 1 lists, in detail, the consensus process by which a road event reported by client X from cluster Y is validated or invalidated. At the client level, the event is encrypted using an encryption key shared between the client and server, and then the encrypted data is sent to the central processing unit. The server receives the encrypted data and decrypts it using the same symmetric key. This data is sent to all clients within the same cluster, Y, encrypted using unique keys, corresponding to each recipient. No information related to the identity of the initiator is disclosed. Each client within the corresponding cluster receives the encrypted data, decrypts it, verifies the veracity of the information and sends its encrypted response to the server. The server collects all the responses from clients and decrypts them. Depending on their answers and the reputation stored on the blockchain, it calculates the percentage of the truthfulness of the information. If the percentage exceeds 50%, the event is considered validated and the blockchain is updated.
**Algorithm 1** Consensus algorithm regarding traffic conditions.Client X from cluster Y:user loads data_trafficencrypted_data = Encrypt (data_traffic, shared_key_X)Send (encrypted_data) to Server
Server:Receive (encrypted_data) from Client Xdata_traffic = Decrypt (encrypted_data, shared_key_X)for each Client[i] from cluster Y except client X
*encrypted_data = Encrypt (data_traffic, shared_key_i)*Send (encrypted_data) to Client[i]end for
Client[i] from cluster Y (except client X):Receive (encrypted_data) from Serverdata_traffic = Decrypt (encrypted_data, shared_key_i)answer = boolean_test (data_traffic)encrypted_answer = Encrypt (answer, shared_key_i)Send (encrypted_answer) to Server
Server:positive_answers = 0negative_answers = 0for each Client[i] from cluster Y except client X
*Receive (encrypted_ answer) from Client[i]*validation = Decrypt (encrypted_ answer, shared_key_i)*if (validation * client_reputation == true)*        positive_answers ++*else*        negative_answers ++*end ifend forif (positive_answers/negative_answers) > 50%
*event = true*update blockchainend if

To validate an event, we considered setting a threshold of 50%. Increasing this threshold implies greater accuracy of traffic data, but the disadvantage is that fewer data will be validated, so the described system will have a limited set of traffic data. Lowering the threshold implies that false data related to traffic can be saved in the system more easily. Let us take an example in which at an intersection there are 10 new clients enrolled in the system, and one of them reports an accident. At this moment the server sends a message to validate this information to the rest of nine clients. Thus considering the threshold of 50%, if five clients validate the information and four invalidate it, it will be saved. If the threshold was 75%, the event would not have been saved.

At this point, we consider that the central server has all the data necessary to accomplish its main purpose: a list of nodes divided into geographic or cluster areas, verified traffic information and the availability of public transport, maps of interest areas. With all this information, clients can receive secure, verified data about the optimal route they can travel from the start location to the destination according to the chosen means of transport. Next, in Algorithm 2, we present the optimal route computation process with more details.
**Algorithm 2** Optimal route computation.Client X:Select preferred means of transport (personal car, shared personal car, public bicycle, taxi, shared taxi, bus, tram, subway, etc.)Insert start location and end locationencrypted_start_location = Encrypt (start_location, shared_key_X)encrypted_end_location = Encrypt (end_location, shared_key_X)encrypted_ preffered_means_of_transport = Encrypt (preffered_means_of_transport, shared_key_X)Send    (   encrypted_start_location,    encrypted_end_location, encrypted_ preffered_means_of_transport) to Server
Server:Receive (   encrypted_start_location,    encrypted_end_location, encrypted_ preffered_means_of_transport) from Client Xstart_location = Decrypt (encrypted_start_location, shared_key_X)end_location = Decrypt (encrypted_end_location, shared_key_X)means_of_transport = Decrypt (encrypted_means_of_transport, shared_key_X)clusters[] = Retrieve_clusters (start_location_cluster, end_location_cluster)for each cluster i from clusters[]//find out the availability of means of transport in each cluster depending on client’s preferences
*M[i] = existing_transport_means && preffered_means_of_transport//M is matrix*for each mean of transport j that exists and is preffered
∘M[i][j] = availability of transport means extracted from blockchain and selected by Client X*end forend forroutes[] = compute_route (M[][])optimal_route = minimum (routes[])encrypted_optimal_route = Encrypt (optimal_route, shared_key_X)Send (encrypted_optimal_route) to Client X
Client X:Receive (encrypted_optimal_route) from Serveroptimal_route = Decrypt (encrypted_optimal_route, shared_key_X)Start to travelfor each cluster i from clusters[]
*for each transport mean j within cluster that client uses
∘enc_msg = Encrypt (availability_of_public_transport_mean, shared_key_X)∘Send (enc_msg) to Server*end forend for

Algorithm 2 presents the process by which the optimal route computation is performed depending on the options chosen by a client, X. Through the client application, the user inserts the main options related to the journey he/she intends to make, options such as start location, end location and preferred means of transport, for example, personal car, bicycle, bus, etc. This information is encrypted using a symmetric key known only by the sender and by the recipient and sent to the server. The server receives the encrypted information, decrypts it and begins to perform the process of identifying the optimal route. The process takes into account all the geographical areas or clusters between start location and end location, the availability of the means of transport, as well as the user’s preferences. Based on these data, all possible transport routes are calculated, the most time convenient route is identified and sent back to the client in an encrypted manner. The client receives the encrypted message, decrypts it and starts travelling. During his/her journey, he/she has the opportunity to contribute traffic data, data that will be sent in encrypted mode to the server.

In such a system, the data regarding the exact location of the clients are very important. Considering that the client application sends GPS data regarding the location to the server, and the GPS has an accuracy of several meters [26], the use of raw data could introduce errors into the system. Thus, considering the server application design, more exactly the Database module in which the clients’ location is saved, the Processing module within the same design can observe certain patterns. With these patterns, customer location information can be refined and thus the proposed system can provide better results.

### Transactions

**Figure 8:**
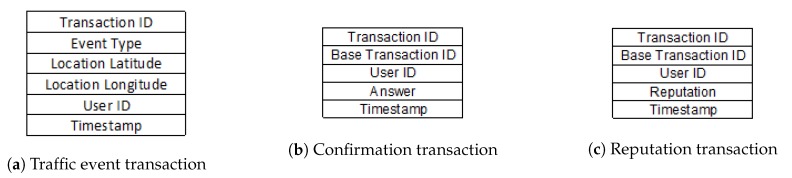
Visual representations of the transaction for traffic event, confirmation and reputation.

As a consequence of finalising the consensus process within a cluster, the system generates a block containing a list of three types of transactions: traffic event transactions, confirmation event transactions and reputation transactions, collectively shown in Figure 8.

The list of transactions within a block always starts with a traffic event transaction. As shown in Figure 8a, this type of transaction contains the following fields. Transaction ID, Event Type, Location Latitude, Location Longitude, User ID and Timestamp. Transaction ID is an integer, unique in the entire blockchain. Within the application are defined a series of road events, such as flood, landslide, blocked traffic, ice on the road, etc.; each of these events has a unique ID assigned. Event Type is a field that stores an ID of the aforementioned. Location Latitude and Location Longitude are GPS coordinates of the user-reported road event, whereas the User ID field represents the ID of the user that reported the event. Timestamp is the datetime field corresponding to the creation of transaction, and implicitly the road event. The creation of this type of transaction is determined by the reporting of a road event by a client application user. This type of transaction is found only once in the list of transactions of a block, at the beginning of the list.

The validity or invalidity of a road event signalled through the type of transaction described above is verified through the consensus algorithm. All nodes within the same geographical area or cluster, represented by the client applications, are queried to confirm the respective event. If a proportion greater than 50% of them confirms the event, this leads to the creation of the block with event information, and the confirmation is represented by the next type of transaction. As shown in Figure 8b, the confirmation transaction contains the following fields. Transaction ID, Base Transaction ID, User ID, Answer and Timestamp. Transaction ID is an integer unique in the entire blockchain, specific to the transaction that confirms the event whose ID is Base Transaction ID. User ID is an integer that represents the ID of the client whom the transaction is due, whereas Answer represents a digit (1/0) through it is stored the user’s opinion regarding the validity of the event (1 = true; 0 = false). In the end, Timestamp is a datetime field corresponding to the creation of the current transaction.

The last type of transaction is reputation transaction. This is created as a result of the validation or invalidation of a traffic event and is stored in the list of transactions within the block related to the event. Its role is to store the reputation of a single client who has contributed to the consensus algorithm. The current reputation of a client is represented by the last transaction within the blockchain; the old transactions within the blockchain can be consulted only to view the client’s historic reputation. If the majority decision is the same as the client’s decision, his/her reputation will increase; otherwise, it will decrease. As shown in Figure 8c, this type of transaction contains the following fields: Transaction ID, Base Transaction ID, User ID, Reputation and Timestamp. Transaction ID is a unique integer in the entire blockchain, specific to the transaction that describes the client’s reputation that validated/invalidated the event which ID is Base Transaction ID. User ID is an integer that represents the client’s ID whose reputation, which is found in Reputation field, is updated. Timestamp represents the transaction’s creation time and can be stored in datetime format.

**Figure 9:**
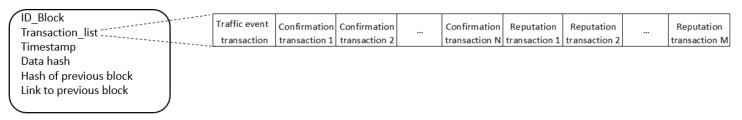
Example of the transactions list within a block.

Figure 9 gives an example of a list of transactions within a block. Creating a block within the blockchain corresponds to the validation or invalidation of a traffic event, and each block has a list of transactions related to the actions and results of this event. The list always starts with a traffic event transaction and after it, there is a number of confirmation transactions and number of transactions that is equal to the number of nodes that validated or invalidated the event. The last type of transaction is reputation transaction, and the number of this type of transaction is equal to the number of confirmation transaction + 1 (the reputation of the client who reported the event is also updated).

Reputation transactions are created internally, at the server level. Nodes do not have direct access to this information, which is useful in calculating the accuracy of road events. However, if an attacker wishes to modify the information within the Transaction list, this attack is quickly revealed through the hash of the current block, which can be found in both the current block and the next one.

**Figure 10:**
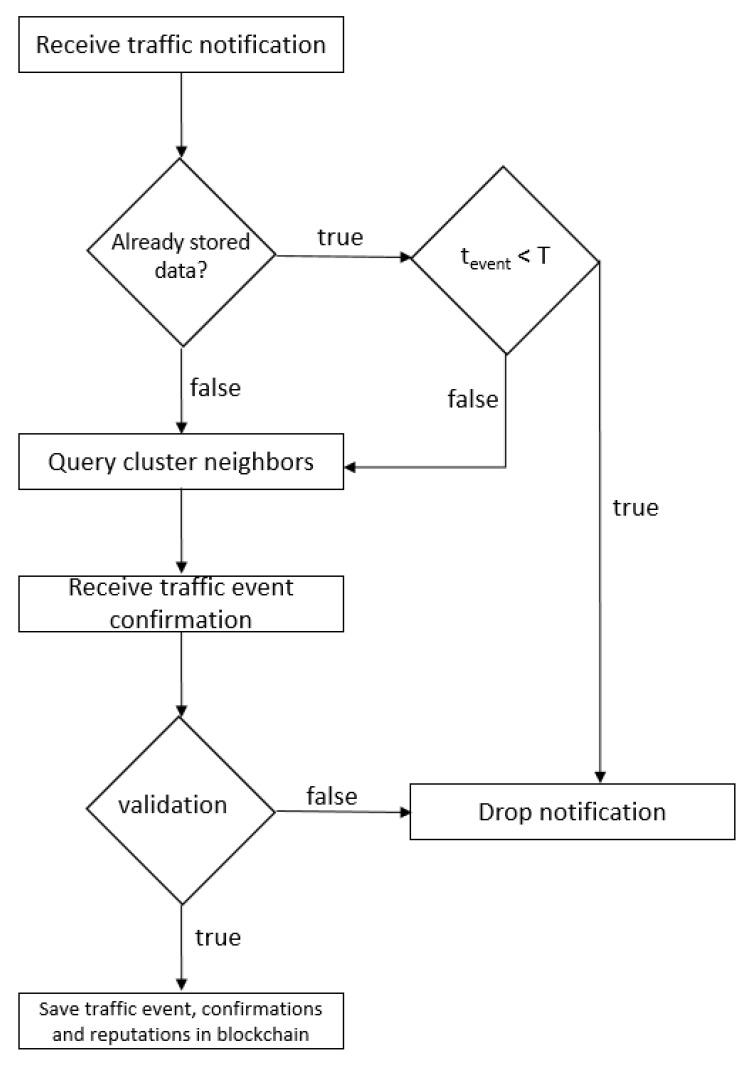
The process of updating ageing data.

The proposed system takes into account the process of data ageing (Figure 10). In traffic, road events can have a relatively short lifespan, so information stored in the blockchain may no longer be current. The client application allows the permanent updating of the data, even if those are permanently stored in the blockchain. Because the deletion of older data is contrary to the principles of blockchain, we chose that this type of update is achieved through the creation of a new type of block that will cancel the information stored in a previous block. Thus, real data within the blockchain will always be the last stored. In this case, the consensus algorithm will be run again after a temporal threshold, as shown in Figure 8c.

The advantage of using blockchain technology in ITS over traditional proposals which do not employ blockchain is that only the information validated by other clients, possibly untrustworthy, within the consensus mechanism is permanently saved. The impossibility of modifying it is realised by the implemented algorithms, and the hash of a block saved in the current block and the next block is proof that the data is not compromised. The reputation of the users that is saved in the blockchain is used in the verification of traffic information and is permanently updated according to the input provided by them. Thus, the system has the ability to adapt and become more refined as it is used.

## 4. Privacy and Security Analysis

The protection of personal data should be the main concern of all the specialists involved in software development. The main purpose is to protect the identity of the clients, to ensure an encrypted communication channel between the entities and to manipulate and store the data in a secure way so that only the authorised processes will be able to access them. A system that protects personal data must also ensure that the identity of the users cannot be revealed by following certain patterns.

The user has at his/her disposal a series of security policies regarding the sharing of his data. Thus, depending on his/her expertise, the user has a number of simplistic policies, such as apply/disable location sharing or apply/disable speed sharing. For experienced users, the application provides a series of more complex policies, such as disable speed sharing if it exceeds a certain threshold, disable location sharing in a certain area, etc. Within the client application, these policies can be found in the Database module. Depending on the policies applied by the client, the data collected by the Sensor module are filtered using the Processing module and transmitted through the Gateway module. Such confidentiality policies do not exist in popular applications and, even if they do exist, it is not guaranteed that the data are not transmitted to the server, taking into account the fact that the vast majority of applications are closed source. However, if the users would not contribute with traffic data, the system would no longer be able to provide information to other users such as the fastest route or time to spend in traffic. The data provided by the clients are used by the server application to know (or discover) the traffic status. Some of the traffic data (accidents, flood on the road, ice on the road, etc.) provided by the clients are sent to other clients in the same cluster or geographical area for validation. Confidential information, such as ID or reputation, will not be sent to other clients. In addition, they have access to the final result offered by the application, such as the optimal route between two locations or the time to spend in traffic.

The only way a user is identified within the system is through a unique ID. Associated with this ID is an encryption key shared only with the server through which the communication between the two entities is encrypted. The symmetric key sharing process is described in the following mathematical model. When it is necessary or at a certain time frame, the encryption key can be changed, so both the communication and the user’s identity are protected. The correspondence between ID and key is known only by the client and the server. The interaction between a client and the other nodes within the network is not direct, the communication is realised through the server. Thus, two cases of communication can be distinguished: signalling an event in traffic and obtaining traffic data. In the first case, a user enrolled in the system with an ID known only by him and the server sends an event to the server. The server receives this information and resends it to the other clients within the same geographical area. The other clients receive only data related to traffic events, not the ID of the client who created the information and if it is validated, the information is saved. The second case is the one in which a user wishes to obtain traffic data, such as the optimal route to travel from point A to point B. He will receive strictly traffic information, not who generated it.

Table 2 presents a simple mathematical model for new user registration, as well as to signal an event in traffic. The operations are described in chronological order according to the entity that performs them: on the left side are the operations that take place at the client application level and on the right side the operations that take place at the server application level.

In the previously mathematical model, one of the security mechanisms proposed within the presented system design is described. Following the National Institute of Standards and Technology (NIST) recommendations [26], we chose to use a mechanism that is based on symmetrical encryption system, as well as on a key exchange system that takes place during the process of registering a new user and during the process of changing the encryption key. In our implementation, we have chosen the well-known Diffie–Hellman algorithm that is used to establish a shared secret key used for encrypted communications over public networks. According to [27], it is proposed to use at least 2048 bits key size. For simplicity’s sake, we chose to present the algorithm in the first six lines using only seven variables. At line six both the client and the server have the secret key used in both encryption and decryption. As the first part of the mathematical model describes the steps to register a new user, in the end, the server will generate a unique ID for the new client and will initiate his/her reputation with 0.

In the second part of the model, the steps through a client transmits to the server a traffic event are presented. At the client application level, the variable related to the type of event is generated, as well as the variables related to the geographical position—latitude and longitude. The data are encrypted using the symmetric key created in the first part of the model and transmitted to the server. At the server application level, those are decrypted, attached a timestamp, and associated with the ID of the client that created them.

## 5. Performance Analysis

In this section, we want to analyse the performance of the proposed approach. The purpose of the tests is to evaluate system characteristics, such as network loading and increasing the blockchain storage size. In the last experiment, using a software application, we simulate an attack in which the malicious nodes manage to validate the fact that it is congestion at 20% of traffic light intersections, following modification of the average speed and fuel consumption.

For this, we have used Sim2Car, a microscopic traffic simulator developed within the University Politehnica of Bucharest [28]. It is based on a realistic mobility model, and also offers API for application development on two layers. The simulator uses street graphs extracted from OpenStreetMap and can simulate thousands of cars in real city environments, so it can be tested with different scenarios under realistic conditions. The basis of these realistic conditions is mobility traces from different cities, including Rome, Beijing and San Francisco.

Given that the purpose of the proposed system design is to promote the development of an application based on the prototype described, its use by a larger number of customers is directly determined by the degree of satisfaction offered. The response time of the application is determined by several factors, one of them being the network loading. The first system performance test consists of analysing the number of messages conveyed within the most important process within the entire system, namely, the consensus algorithm, through which the veracity of traffic information is established.

**Figure 11:**
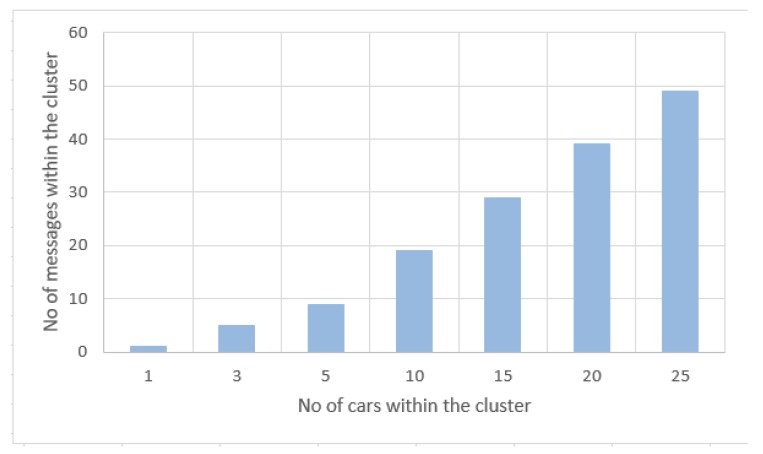
No of messages within the cluster/no of cars within the cluster.

Table 3 and Figure 11 presents the test results that perform the correlation between the number of cars within the cluster and the number of messages within the geographical area transmitted in the network to validate/invalidate a traffic event. On the OX axis is the number of cars or nodes within the cluster, and the OY axis represents the number of messages. The messages represent the two types of transactions that transit the network: traffic event transactions and confirmation transactions. This exchange of messages within the network is realised between the nodes represented by cars and the main entity of the system design, the central server. In the presented communication workflow the number of messages is minimised; thus, the first message generated is the one through which a road event is signalled, is sent by a node within a cluster to the server. The rest of the messages are transmitted between the server and the other nodes within the same cluster, pair messages of the type: *is the event X valid?* and *yes/no*. The communication workflow is lightweight, and the network is not loaded with duplicate or useless messages. Therefore, the relation between the number of messages and the number of cars within the cluster is y=1+(x−1)∗2, where *y* represents the number of messages and *x* represents the number of cars.

**Figure 12:**
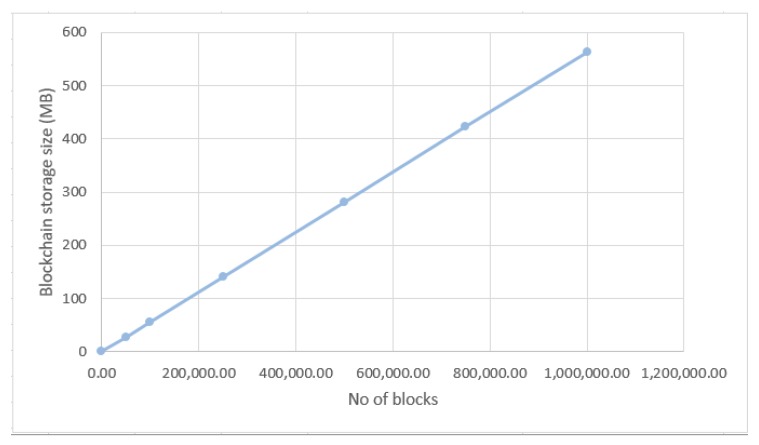
Blockchain storage size based on the number of blocks.

The second system performance test consists of analysing the increase of the blockchain storage size according to the number of blocks. The creation of a new block in the blockchain takes place in two cases: the validation of a traffic event or the invalidation of an event already stored, but which is no longer valid. The first case is specific to the situation in which some node or user of the client application signals to the server a road event in a certain geographical area, also known as a cluster. At this point, the server interrogates all other clients in the same cluster and if the possibility of the event to be true is greater than 50%, then the event is saved in the blockchain as a new block. The second case is specific to the situation in which the system warns clients about a particular event. They invalidate the event and, if the possibility of the event to be true is greater than 50%, the event is invalidated creating a new block within the blockchain; also called consensus algorithm, and through it, the server can realise the real traffic situation. The standard size of a node without the transaction list is 64 bytes. We suppose that a number of 10 cars participate in the validation or invalidation of a road event. Thus, the list of transactions consists of one traffic event transaction, nine confirmation transactions, and 10 reputation transactions. Thus, the size of a block increases to 564 bytes. In Table 4 and Figure 12 it is presented the increase of the blockchain storage size according to the number of blocks, considering an interval from 0 to 1,000,000 blocks. On the OX axis is the number of blocks and the OY axis represents the storage size in megabytes (MB). According to the graph below, for a number of 1,000,000 blocks a storage space of 564 MB is required. Considering that the stored traffic data is validated and the system also saves the user reputation that is taken into account for future transactions, we can conclude that such a concept implemented in real life using appropriate technical infrastructure (high-performance servers) will have good results.

In the third test, we used Sim2Car to simulate traffic conditions under two different conditions: intelligent routing without malicious nodes that managed to store false data into the blockchain, and intelligent routing where malicious nodes managed to insert into the blockchain the following event; congestion at 20% of traffic light intersections. The purpose of attackers to insert fake data into the blockchain is to release certain routes for their benefit. The results of this test for all three cities are presented in Table 5. Comparing the results mentioned above, the average speed of all cars decreased by 1.16% for Rome, 8.29% for Beijing and 6.96% for San Francisco. In terms of average car consumption, it decreased by 0.96% for Rome, 3.23% for Beijing and 2.1% for San Francisco.

**Figure 13:**
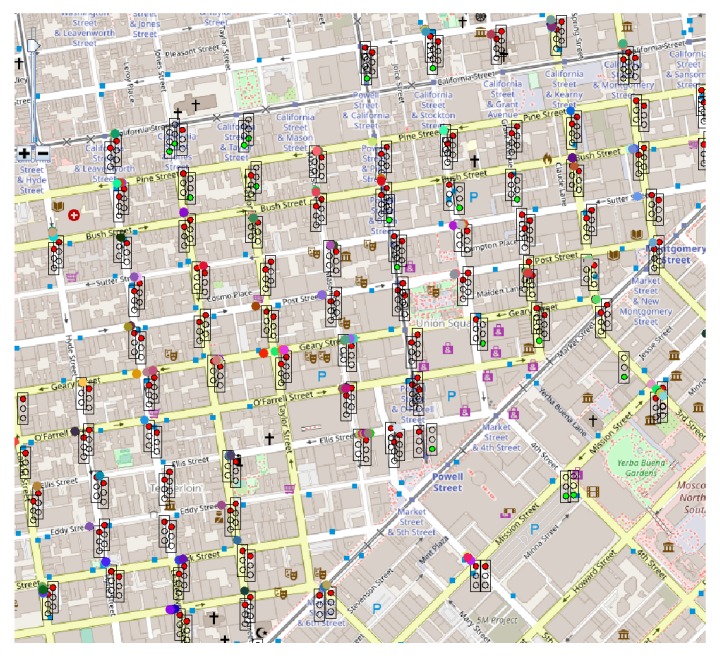
Sim2Car screenshot for traffic conditions in San Francisco.

**Figure 14:**
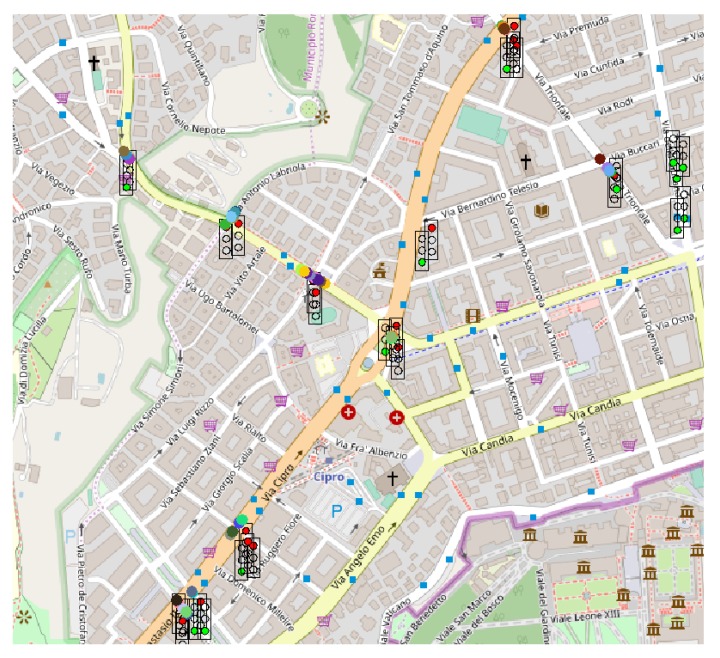
Sim2Car screenshot for traffic conditions in Rome.

**Figure 15:**
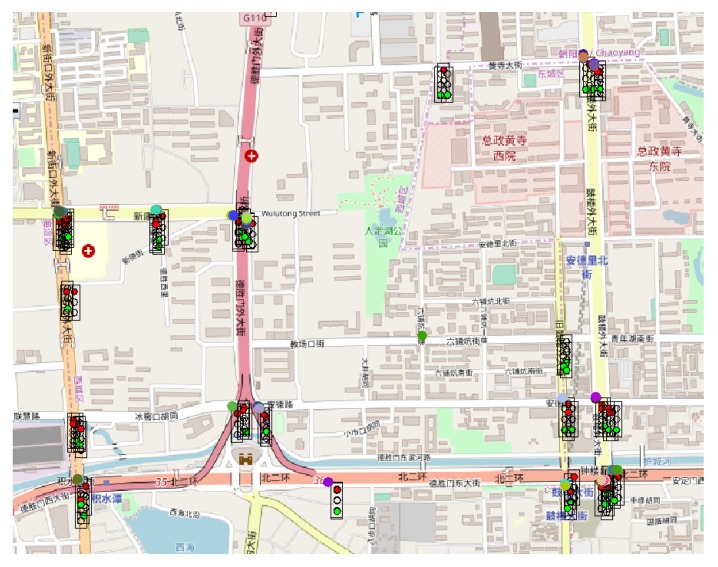
Sim2Car screenshot for traffic conditions in Beijing.

Through the graphical interface available in Sim2Car the traffic situation can be observed in real-time. Figure 13, Figure 14 and Figure 15 show screenshots of traffic conditions in San Francisco, Rome and Beijing, respectively. All three cases are valid for the situation where malicious nodes managed to insert the fact that it is congestion at 20% of traffic light intersections. As we can see in the images mentioned above, there are several busy intersections with a significant number of cars that are waiting in line. This fact determines the decrease in the average speed of the vehicles and at the same time the decrease of fuel consumption, results mentioned above. As the “good” nodes came in crowded clusters or geographical areas, signal these congestions to the system, and the other “good” nodes guarantee these events, the congestions will disappear by routing the cars to less crowded areas.

Depending on the simulation results, we can conclude the following.

- For all three cities, the average speed decreased if erroneous information was stored in the blockchain as an implemented routing algorithm guides the honest cars on other free routes and thus crowds other intersections.
- For all three cities, the average consumption decreased if incorrect information was stored in the blockchain. This is due to the reduction of the average speed of the vehicles.
- Both average speed and average consumption are closely related to the information stored in the blockchain, information validated within the consensus algorithm. Therefore, for the data stored to reflect the actual traffic conditions, it is demonstrated that it must be validated by the rest of the users and, in addition, the user’s reputation must be taken into account. Finally, even if an attacker manages to store false data into the blockchain, it will soon be invalidated by other traffic participants under a new consensus mechanism.

## 6. Conclusion

In this paper, we presented a reputation system used in Intelligent Transportation System where privacy-preserving is our main concern. Our proposal is based on blockchain that can reach a consensus within a decentralised network potentially made up of unreliable nodes. The main actors are vehicles and a central authority responsible for blockchain storage and trust computation. The communication is made only between the vehicles and the central server in an encrypted manner and the consensus process is carried out between all the vehicles in the same cluster or geographical area. The result of the consensus algorithm is the validation or invalidation of road events, as well as updating the participants’ reputation. These are stored securely and permanently in the blockchain and cannot be modified. The system also takes into account the ageing process of road data after a certain threshold. The final purpose of the presented system is to provide to users an optimal travel route based on reliable data, as well as maintaining confidentiality.

Our future research direction focuses on accurately identifying the GPS position of users considering more weights, such as patterns. Also, more experiments can be performed to improve the proposed system design.

## Figures and Tables

**Table 1:** Comparative analysis of related work.

Reference Work	Advantages and Disadvantages		
	Guaranteed Confidentiality	Use Reputation Metric in Traffic Event Validation	Easy Implementation in Real-Life Scenario
[21]	no	no	yes
[20]	no	no	yes
[22]	no	no	yes
[23]	no	yes	no
[24]	yes	yes	no
[25]	yes	no	no
**OUR PROPOSAL**	yes	yes	yes

**Table 2:** Mathematical model describing the operations used to register a new user and to signal an event in traffic.

Row No.	Client		Server
Register a new user			
1	**g** –small prime number	→	
2		←	**n** –large prime number
3	Generate **a**		Generate **b**
4	A = g^a^ mod n	→	
5		←	B = g^b^ mod n
6	s = B^a^ mod n	=	s = A^b^ mod n
7		←	Generate uID_k_ ∉ {uID_1_, uID_2_, uID_3_, ...}
8			Initialization r_k_ = 0
Signal a traffic event			
9	Generate **eventID**		
10	Generate **GPS_latitude**		
11	Generate **GPS_longitude**		
12	**msg**=(eventID; GPS_latitude; GPS_longitude)		
13	**enc**=Encrypt(msg; s)	→	
14			**msg** = Decrypt(enc; s)
15			Generate **t** –timestamp for msg
16			**event** = (msg, t, uID_k_, r_k_)

**Table 3:** Experimental results: Analysis of the number of messages within the cluster according to no of cars within the cluster.

No of Cars within the Cluster	No of Messages
1	1
3	5
5	9
10	19
15	29
20	39
25	49

**Table 4:** Experimental results: Analysis of the blockchain storage size according to the number of blocks.

No of Blocks	Total Storage Size (bytes)
1	564
50.000	28.200.000
100.000	56.400.000
250.000	141.000.000
500.000	282.000.000
750.000	423.000.000
1.000.000	564.000.000

**Table 5:** Experimental results: average speed and fuel consumption.

City	Normal Traffic		20% Blocked Intersections	
	Avg Speed [km/h]	Avg Fuel [l/h]	Avg Speed [km/h]	Avg Fuel [l/h]
Rome	41.24	11.49	40.76	11.38
Beijing	46.27	11.43	42.43	11.06
San Francisco	41.09	11.4	38.23	11.16

## Abbreviations

The following abbreviations are used in this manuscript:

CA	Certificate Authority
GPS	Global Positioning System
IEEE	Institute of Electrical and Electronics Engineers
IoT	Internet of Things
ITS	Intelligent Transportation Systems
LEA	Law Enforcement Authority
NIST	National Institute of Standards and Technology
OBU	On-Board Units
RSU	Roadside Units
VANET	Vehicular Ad Hoc Network
V2V	Vehicle-to-Vehicle
V2I	Vehicle-to-Infrastructure
